# Seroprevalence data at a private teaching hospital in Kenya: An examination of *Toxoplasma gondii*, cytomegalovirus, rubella, hepatitis A, and *Entamoeba histolytica*

**DOI:** 10.1371/journal.pone.0204867

**Published:** 2018-10-16

**Authors:** Audrey I. Nisbet, Geoffrey Omuse, Gunturu Revathi, Rodney D. Adam

**Affiliations:** 1 University of Arizona College of Medicine, Tucson, Arizona, United States of America; 2 Department of Pathology, Aga Khan University Hospital, Nairobi, Kenya; 3 Department of Medicine, Aga Khan University Hospital, Nairobi, Kenya; Gettysburg College, UNITED STATES

## Abstract

**Background:**

Relevant seroprevalence data for endemic pathogens in a given region provide insight not only into a population’s susceptibility to acute infection or risk for reactivation disease but also into the potential need for policy initiatives aimed at reducing these risks. Data from sub-Saharan Africa are sparse and since Aga Khan University Hospital Nairobi is an internationally accredited hospital equipped with a laboratory electronic medical record system, analysis of pertinent local seroprevalence data has been made possible.

**Methods:**

We have analyzed serology data from laboratory electronic records at a 300 bed tertiary private teaching hospital in Kenya for the dates, 2008 to 2017 for *Toxoplasma gondii*, cytomegalovirus, and rubella, which were used primarily for antenatal screening. We also analyzed the data from hepatitis A and amebiasis serologies, which were used primarily for diagnostic purposes.

**Results:**

For *T*. *gondii*, cytomegalovirus, and rubella, we used IgG serology to determine seroprevalence, finding rates of 32%, 86%, and 89%, respectively. There was no significant age-related difference in the 20 to 49 year old age range for any of these three pathogens. Of the Hepatitis A IgM tests that were ordered, 33% were positive with a peak positive rate of 70% in the five to nine year old age range. The seroprevalence of amebiasis was 4% and all cases of seropositivity were accompanied by compatible clinical illness (hepatic abscess).

**Conclusions:**

These data provide insight into seroprevalence rates of selected pathogens that can be used to guide screening and diagnostic laboratory testing as well as private and public immunization practices.

## Introduction

Seroprevalence data can provide information useful for clinicians when it comes to timely initial empirical treatment, patient education, and evaluation of susceptibility to acute infection or risk for reactivation disease in vulnerable patient populations. These data can also be leveraged by public health officials and policy makers during the formulation of vaccination guidelines, patient screening practices, and health communication initiatives.

The specific significance of each of the five pathogens presented in this report varies in terms of their application to clinical practice and policy. There are certain infections where the knowledge of local seroprevalence data is useful for informing clinical decision making. The great majority of *Toxoplasma gondii*, rubella, and CMV serologies at the Aga Khan University Hospital Nairobi (AKUHN) are done as part of prenatal screening. Thus, the seroprevalence for these entities is likely to reflect the local seroprevalence in young adults.

The specific applications of seroprevalence data depend on the pathogen being considered. For rubella, the major morbidity is congenital rubella syndrome. Thus, the seroprevalence in the general population as well as in women of childbearing age will directly impact the potential risk for congenital rubella syndrome. Seropositivity can result from either immunization or from natural infection and an immunization rate of over 95% is considered an adequate population level. In Kenya and many other developing countries that do not have longstanding rubella immunization programs, the infection rate during childhood is high but whether that rate is high enough to provide population level protection needs to be determined.

Cytomegalovirus (CMV) is also of concern particularly because of its association with congenital infection. Children born with CMV infection may have systemic infection and/or congenital abnormalities at birth or may have neurologic manifestations that occur after birth. There is now evidence that at least some of these congenital infections should be treated [1]. The greatest risk of congenital CMV occurs when the mother’s primary CMV infection occurs during pregnancy, but can also occur as a result of reactivation or intermittent viremia during pregnancy [2]. In high prevalence areas, the majority of congenital infections may occur as a result of maternal reactivation rather than primary infection simply because there are many more mothers at risk for reactivation than primary disease. HIV-infected mothers with advanced HIV infection may be at higher risk because CMV viremia is common in these patients [3]. Globally, rates vary substantially with adult seroprevalence rates varying from about 50% to nearly 100% [4].

Infection with *T*. *gondii* is acquired primarily by exposure to newly infected cats or by eating infected meat and unwashed fruits and vegetables and sometimes drinking water contaminated by cat feces [5, 6]. However, vertical infection can also occur when the mother becomes infected during pregnancy and may result in congenital infection with neurologic manifestations that present in the newborn or develop in childhood and adolescence. After primary infection, the individual develops a latent infection that lasts for life. Because of this latent infection, reactivation disease can occur in immunocompromised patients, especially in advanced HIV infection [7] and in transplant recipients.

Hepatitis A is the most common viral cause of acute hepatitis globally [8] and disease varies with seroprevalence. In high prevalence areas, most infections occur during early childhood when the infection is typically asymptomatic or subclinical, so symptomatic disease is relatively uncommon. However, as seroprevalence rates drop and the age of acquisition increases, more infections occur during late childhood or adulthood with an increased number of hepatitis cases. Then, as prevalence drops further as occurs in many high income countries, the disease rate drops, and even more so when immunization becomes common. Thus, knowing age-specific prevalence data helps to determine local risk of disease.

*Entamoeba histolytica* is a protozoan parasite that is transmitted by food or water and causes colonic infection with acute dysentery as well as extra-intestinal invasive disease, particularly liver abscess [9, 10]. Accurate morphologic diagnosis by stool examination is impossible because the nonpathogenic organisms, *Entamoeba dispar* and *Entamoeba moshkovskii*, are commonly found and cannot be distinguished morphologically from *E*. *histolytica*. In many settings, these organisms are found much more frequently in fecal samples than *E*. *histolytica*. Amebic serology is positive in about 80–90% of patients with amebic colitis and greater than 95% in those with amebic liver abscess [11]. In addition, the specificity is quite high in most settings, making serologic testing very useful for the diagnosis of invasive amebiasis. There are some reports of a high background seroprevalence rate in highly endemic regions (e.g. 5–10%) [11], which would lower the specificity and clinical usefulness in those settings. Thus, an estimate of the background seroprevalence rates will aid in the interpretation of a positive serologic result.

Seroprevalence data from Kenya and the remainder of sub-Saharan Africa are sparse and some of the available publications are from decades ago and may not reflect current rates. Thus, we have analyzed seroprevalence data for these five pathogens from the AKUHN clinical laboratory.

## Materials and methods

AKUHN is a 300 bed university hospital in Nairobi Kenya with a range of post-graduate medical education programs. The patients are primarily from the upper middle and high socioeconomic groups comprised mainly of black African Kenyans, Kenyans of Asian descent, and a small Caucasian population ‒ mostly expatriates. The moderate numbers of HIV-infected patients seen at the hospital reflect the national HIV prevalence of nearly 7%, which is midrange for sub-Saharan Africa. The hospital has about 50 critical beds, including intensive care unit, coronary care unit, cardiothoracic intensive care unit, neonatal intensive care unit, and high dependency units. There is a nine bed outpatient dialysis unit and a cancer center. The hospital conducts approximately 3600 deliveries in a year. The laboratory was the first hospital laboratory in East Africa to become internationally accredited by ISO 15189 standards through the South Africa national accreditation service (SANAS) [12]. The Aga Khan hospitals in Mombasa and Kisumu have bed capacities of approximately 80. The laboratories at these hospitals are also accredited by SANAS and refer their more complex tests, including serologies to the Nairobi facility.

### Antibody testing

Amoebic serological testing was routinely performed in the laboratory using a commercial indirect hemagglutination assay (Cellognost–Amoebiasis IHA (Siemens Healthcare Diagnostics Products GmbH, Marburg, Germany) that allows qualitative and quantitative antibody detection. The assay uses human RBCs coated with antigenic extract of axenically cultured parasites.

Quantitative determination of IgG antibodies for CMV, *T*. *gondii* and rubella was performed on a Cobas e601 analyzer (Roche diagnostic GmbH, Mannheim, Germany) that uses the electro-chemiluminescence immunoassay (ECLIA) principle. Amebiasis serology was carried out using a Cellognost kit (Siemens Healthcare, Diagnostics products GmbH, Marburg, Germany). All these tests were enrolled for the Randox International Quality Assessment Scheme (RIQAS) external quality assurance (EQA) program with satisfactory performance during the period of interest.

### Data analysis

Serology data from 2008 through 2017 were extracted from the laboratory electronic medical record system. Available information included date of test, date of birth, patient sex, outpatient/inpatient status of the patient, admit date (if inpatient), and specific hospital, clinic, or ward in which the order was requested.

When repeat orders were listed for a single source, only the earliest completed order was included in the analysis. Patients under one year of age were excluded, as these samples could represent maternal acquired antibodies and may not accurately reflect exposure to or infection with a given pathogen. Patients listed as 99 years of age were also excluded from analysis because of the suspiciously large number of entries with this age. It is our speculation that an age of 99 years was assigned in the absence of accurate birth date information.

Data analysis for *T*. *gondii*, cytomegalovirus, and rubella focused on IgG antibody serology collected in the outpatient setting with the objective of determining seroprevalence in young adults in the population served by this hospital. The vast majority of these tests are ordered for prenatal care, given the particular significance of these pathogens for fetal health, and are therefore likely good indicators of seroprevalence in young adults. The total sample set was analyzed based on the above criteria, and when sample size permitted, regional analysis for three cities of interest; Nairobi, Mombasa, and Kisumu, was also completed. Age analysis in ten-year increments was performed for the total sample, as well as for Nairobi, when the sample size was large enough. Finally, analysis by sex was done for the total sample set, for Nairobi, and by age, when possible.

Hepatitis A serologic tests are most commonly ordered as IgM test only, but may also be ordered as total plus IgM. We analyzed IgM results to determine how common Hepatitis A was when the diagnosis was suspected and, for those with negative IgM results, analyzed the seroprevalence for total antibody to estimate the background seroprevalence. Finally, both these sets of data were examined by age in five-year increments.

Our goal with amebiasis was to analyze percent positivity and correlation with clinical illness in an effort to determine the specificity of serology orders completed at AKUHN, as well as background prevalence of amebiasis within the institution’s patient population. To determine specificity and background prevalence of amebiasis, all sources with positive serology results were assessed for clinical illness by evaluation of radiological reports, with the goal of determining whether or not there was evidence of hepatic abscess.

For descriptive statistics, percentages are presented with their corresponding 95% confidence intervals. For inferential statistics, a p value less than 0.05 was considered statistically significant.

### Ethics review

The study was reviewed and approved by the clinical research ethics committee of Aga Khan University Nairobi, Kenya. All individual patient-identifying information was removed prior to final analysis and as such, the study was granted a waiver of requirement for individual consent by the ethics review committee.

## Results

### T. gondii

The *T*. *gondii* IgG seroprevalence rate is relevant in determining the risk for vertical transmission during pregnancy, since vertical transmission is largely limited to women who seroconvert during pregnancy [13]. It is also relevant for empiric treatment of space-occupying brain lesions in advanced HIV infection, since *T*. *gondii* is rare as a cause of space-occupying cerebral lesions in HIV patients who are seronegative for *T*. *gondii*. Thus, a negative serologic result can be used to eliminate toxoplasmosis from active consideration in seronegative patients [14, 15]. Analysis of the total number of toxoplasma IgG serology samples revealed a seroprevalence of 32% (n = 1383). The availability of specimens from sister hospitals in the Indian Ocean city of Mombasa and the Lake Victoria city of Kisumu made it possible to compare prevalence rates in these two cities with that of Nairobi (Table 1). All three hospitals care primarily for middle to upper class patients, so these rates may not reflect the rates seen in lower classes or in rural Kenyans. It is notable that the *T*. *gondii* seroprevalence rate of 27% (n = 1023) in Nairobi is significantly lower than those of Kisumu (52%; n = 44) and Mombasa (57%; n = 160). Additional analysis by age was completed for Nairobi, revealing that most tests were ordered for females aged 20 to 49 years, consistent with the primary use of the test for antenatal screening (Table 2).

**Table 1:** Regional Analysis for *T*. *gondii*.

Location	% Positive (95% CI)
Kisumu (n = 44)	52%
Mombasa (n = 160)	57%
Nairobi (n = 1023)	27%

Nairobi vs. Kisumu: Fisher’s exact two-tailed P = 0.0005

Nairobi vs. Mombasa: Fisher’s exact two-tailed P < 0.0001

**Table 2:** Age Analysis in Nairobi for *T*. *gondii* (age 1 to 49).

Age	% Positive
1 to 9	0% (n = 7)
10 to 19	22% (n = 9)
20 to 29	30% (n = 271)
30 to 39	24% (n = 611)
40 to 49	37% (n = 104)

### Cytomegalovirus

The seroprevalence rate for CMV IgG is 85% for women from 20 to 49 years of age (n = 416), while there is an 86% prevalence rate for all patients (n = 645). When examining the data based on region, we found a statistically significant difference between seroprevalence rates in Mombasa (98%; n = 88) and Nairobi (82%; n = 485) (Fisher’s exact two-tailed P < 0.0001).

### Rubella

The rubella IgG seropositivity rate of 89% (n = 2383) results from a combination of natural infection and immunization. The relative frequency is not known, since the rubella vaccine is frequently administered in the private sector, but it was not included in the government immunization program until a large catch-up campaign in 2016 with plans to include the measles-rubella vaccine as a government sponsored routine immunization the following year. Of note, the majority of tests were ordered for women of childbearing age, presumably for prenatal screening purposes. Female samples (n = 2383) comprised 97% of the total, with the vast majority of women falling between 20 to 49 years of age (n = 2351). Based on this report, approximately 10–12% of women of childbearing age are at risk of rubella infection (Table 3).

**Table 3:** Rubella seroprevalence by age for females aged 20 to 49.

Age	% Positive
20 to 29	88% (n = 798)
30 to 39	89% (n = 1408)
40 to 49	90% (n = 145)
Total	89% (n = 2351)

### Hepatitis A

**Fig 1:**
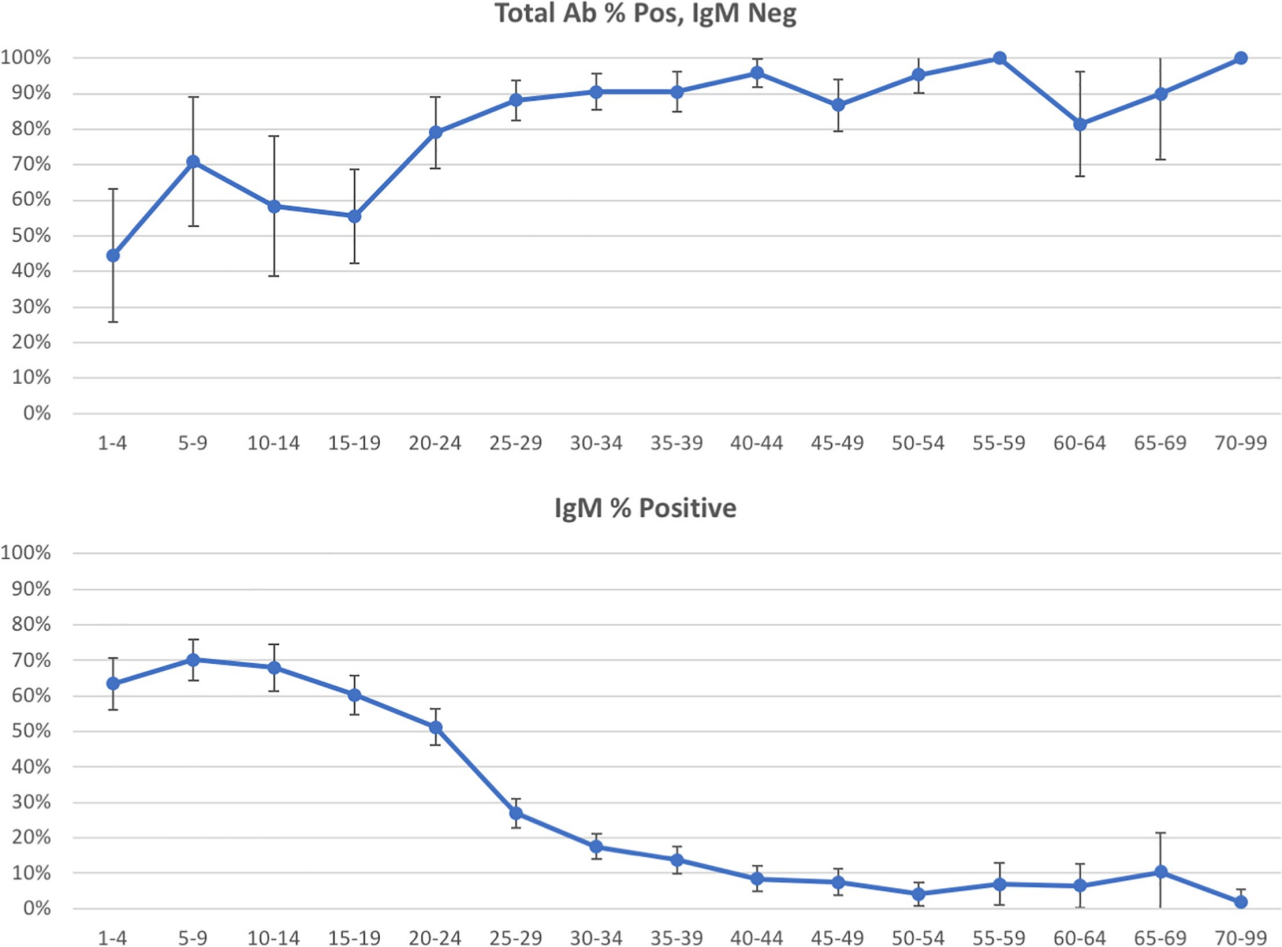
Age analysis for Hepatitis A antibody. The top panel shows the percent of positive tests by age category in five year increments. Age less than one year is omitted because of small numbers and the potential for maternal antibody to affect the total antibody results. All patients above 70 years are grouped together because of small numbers. The lower panel consists of the patients who had both IgM and total antibody performed and whose IgM was negative. The error bars demonstrate the 95% confidence intervals.

Of the Hepatitis A IgM antibody serology tests that were ordered (n = 3265), the positivity rate was 33%. For those orders that were IgM negative (n = 893), 85% were positive for total antibody, indicating an 85% seroprevalence rate for Hepatitis A. A breakdown by age is presented in Table 4 and Fig 1.

**Table 4:** Age analysis for Hepatitis A antibody.

Age	Acute infection (IgM positive)		Past infection (Total Ab positive, IgM negative)	
	Total	% Positive	Total	% Positive
1 to 4	167	63%	27	12 (44%)
5 to 9	251	70%	24	17 (71%)
10 to 14	190	68%	24	14 (58%)
15 to 19	314	60%	54	30 (56%)
20 to 24	377	51%	62	49 (79%)
25 to 29	444	27%	127	112 (88%)
30 to 34	417	18%	126	114 (90%)
35 to 39	312	14%	105	95 (90%)
40 to 44	238	8%	96	92 (96%)
45 to 49	188	7%	83	72 (87%)
50 to 54	148	4%	64	61 (95%)
55 to 59	72	7%	35	35 (100%)
60 to 64	62	6%	27	22 (81%)
65 to 69	29	10%	10	9 (90%)
70 to 99	54	2%	28	28 (100%)

For past infection: Age 1 to 19 (57% positive; n = 129) vs. 20 to 99 (90% positive; n = 763); Fisher’s exact two-tailed P < 0.0001

### Amebiasis

Of 69 amebiasis serology tests performed, three were positive, representing a positivity rate of 4% (0–9%, 95% CI). To determine specificity and background prevalence of amebiasis, clinical illness was assessed in all sources in patients with positive serology results by evaluation of radiological reports, with the goal of determining whether or not there was evidence of hepatic abscess. All three had hepatic abscesses identified by radiological examination, indicating that the amebiasis serology tests achieved a specificity of 100%. Because our sample set did not include any sources that had a positive serology in conjunction with an absence of clinical illness per radiological evidence, we consider our background prevalence of amebiasis to be 0% (0–5%, 95% CI).

## Discussion

Seroprevalence can be estimated from serologic tests performed as part of routine prenatal care, which is the case for the majority of *T*. *gondii*, CMV, and rubella serologic tests. *T*. *gondii* seroprevalence differs widely among countries and areas within countries because of climate and multiple human factors. An earlier study on sera from blood donors in four areas of Kenya showed an overall antibody prevalence of 54%, ranging from 43% to 65% in the four areas [16]. Their rate of 45% from Nairobi is higher than our rate of 27% and could reflect a change in seroprevalence over the last three decades or test methodologies. However, we also note that the patients seen at AKUHN are from middle and upper socioeconomic levels and are likely to have lower seroprevalence rates for these pathogens than those from lower socioeconomic groups. The three Aga Khan hospitals represented in this study care for similar patient populations, allowing for a geographical comparison among Kisumu on Lake Victoria, Mombasa on the Indian Ocean, and Nairobi in a central high elevation area. The 27% positive rate in Nairobi is significantly lower than the rates of 52% and 57% we found in Kisumu and Mombasa, respectively. Feral cats are common throughout Kenya and ingestion of undercooked meat is uncommon, suggesting that most human *T*. *gondii* infection results from inadvertent ingestion of oocysts passed by feral cats. This could be due to direct contact with contaminated soil or from the ingestion of fruits and vegetables contaminated with oocysts [5]. In addition, infection from drinking water is also possible [17]. Perhaps the reason for the lower seroprevalence in Nairobi is because of a lower humidity, making it less suitable for oocyst maturation after being passed in cat feces. Understanding of local seroprevalence rates for *T*. *gondii* is very useful for guiding the approach to HIV-infected patients with space-occupying lesions in the brain [6]. These lesions are nearly always due to reactivation of latent infection; thus, a negative IgG antibody at the time of presentation nearly rules out toxoplasmosis as the diagnosis [18].

Examination of global cytomegalovirus trends amongst women of childbearing age revealed seroprevalence rates from 45 to 100%, with the highest rates observed in South America, Africa, and Asia [19]. Previous analysis of cytomegalovirus seroprevalence in blood donors in Nairobi published in 2009 found an IgG seroprevalence rate of 97.0% [20]. Cytomegalovirus maintains important clinical implications in the maternal population, due to the possibility of vertical transmission to the fetus. The risk is markedly elevated in women who develop their primary infection during pregnancy. However, there is also some risk for transmission by reactivation in women whose primary infection occurred before the pregnancy. In areas with high seroprevalence rates, congenital infection occurring by reactivation can be the predominant form of transmission [2]. We found a somewhat lower seroprevalence rate of 86% in our total sample set and 82% for Nairobi, which could reflect the middle to high socioeconomic composition of our institution’s patient population. However, the seroprevalence rate is still high enough that the bulk of the burden of congenital CMV disease may come from reactivation rather than acute infection during pregnancy.

It is likely that the rubella seroprevalence of 89% in the current report is from a combination of naturally occurring rubella exposure and from prior immunization. Rubella vaccination is frequently given in the private sector, but was not included in the government immunization program, which delivers the majority of immunizations, until a large catch-up campaign in 2016 with plans to include the measles-rubella vaccine as a government sponsored routine immunization the following year. Regardless of the modality of acquired immunity, the prevalence rate of 89% poses a significant susceptibility risk to women of childbearing age with its concomitant risk of congenital rubella syndrome. Our data indicate that approximately 10–12% of women of childbearing age are at risk of rubella infection, a figure that falls well above the 5% rubella susceptibility maximum for women of childbearing age recommended by the World Health Organization [21]. These susceptibility data, in conjunction with WHO recommendations, provide strong evidence to support the inclusion of the rubella vaccine in the national immunization program in Kenya, in an effort to curb congenital rubella syndrome and its potentially devastating effects. Additional support for the provision of a government sponsored rubella vaccine can be found in data collected from the United States, which reflect a 95.3% seroprevalence rate–a value that is attributed primarily to the practice of immunization [22].

Sub-Saharan Africa is home to some of the highest seroprevalence rates of Hepatitis A in the world, with most older children and adults acquiring immunity through natural exposure [8]. A prevalence of 63.2% was found among Kenyan schoolchildren in a study conducted from 1998–1999 [23]. This prevalence is similar to the rate of 57% found in our 1–19 age group.

All positive amebiasis serology results obtained at our institution were accompanied by clinical illness as evidenced by hepatic abscesses found on radiologic examination. The absence of a background prevalence for amebiasis suggests significant value in a positive serology result in a patient with suspected amebiasis. Therefore, positive serology results should be considered a reliable means of confirming amebiasis infection. This is an important finding, as textbooks commonly quote high background seroprevalence rates for *Entamoeba histolytica* infection, which can make a positive test result less useful [11]. The current study indicates that the background prevalence for positive amebic serology is low enough that a positive test is generally associated with amebic disease.

## Conclusion

The information presented in this report can be used by clinicians and policy makers in the continued advancement of healthcare in Kenya. Similarities and differences between the data in this report and existing data for Kenya, other African countries, and regions outside of Africa that have been highlighted here provide sound points of comparison and a foundation upon which an examination of current practices in Kenya can be made. It is also worth noting that for these and other pathogens, age of acquisition increases and seroprevalence rates decrease with increasing income. Thus, it is possible that these seroprevalence rates would be higher if lower income patients were included. Continued evaluation of seroprevalence data and the factors that contribute to these data would be a valuable step in continuing to advance the health of Kenya.

## Supporting information

S1 Table Individual patient results with identifying information removed. (XLSX)
